# mmu-miR-145a-5p Accelerates Diabetic Wound Healing by Promoting Macrophage Polarization Toward the M2 Phenotype

**DOI:** 10.3389/fmed.2021.775523

**Published:** 2021-12-21

**Authors:** Yanhui Hao, Leilei Yang, Ying Liu, Yumeng Ye, Jiayu Wang, Chao Yu, Hua Yan, Yuan Xing, Zhaoqian Jia, Cuicui Hu, Hongyan Zuo, Yang Li

**Affiliations:** ^1^Beijing Institute of Radiation Medicine, Academy of Military Medical Sciences (AMMS), Beijing, China; ^2^Department of Basic Medicine, Chengde Medical College, Chengde, China; ^3^Academy of Life Sciences, Anhui Medical University, Hefei, China

**Keywords:** mmu-miR-145a-5p, diabetes, wound healing, macrophage, inflammation

## Abstract

Diabetic wounds are recalcitrant to healing. One of the important characteristics of diabetic trauma is impaired macrophage polarization with an excessive inflammatory response. Many studies have described the important regulatory roles of microRNAs (miRNAs) in macrophage differentiation and polarization. However, the differentially expressed miRNAs involved in wound healing and their effects on diabetic wounds remain to be further explored. In this study, we first identified differentially expressed miRNAs in the inflammation, tissue formation and reconstruction phases in wound healing using Illumina sequencing and RT-qPCR techniques. Thereafter, the expression of *musculus* (mmu)-miR-145a-5p (“miR-145a-5p” for short) in excisional wounds of diabetic mice was identified. Finally, expression of miR-145a-5p was measured to determine its effects on macrophage polarization in murine RAW 264.7 macrophage cells and wound healing in diabetic mice. We identified differentially expressed miRNAs at different stages of wound healing, ten of which were further confirmed by RT-qPCR. Expression of miR-145a-5p in diabetic wounds was downregulated during the tissue formation stage. Furthermore, we observed that miR-145a-5p blocked M1 macrophage polarization while promoting M2 phenotype activation *in vitro*. Administration of miR-145a-5p mimics during initiation of the repair phase significantly accelerated wound healing in db/db diabetic mice. In conclusion, our findings suggest that rectifying macrophage function using miR-145a-5p overexpression accelerates diabetic chronic wound healing.

## Introduction

Wound healing is a complex process involving a variety of cell types and the coordination of various cytokines to regulate wound repair. Macrophages tightly participate in and regulate the entire process of wound healing, and their numbers increase during the inflammation phase, peak during the tissue formation phase, and gradually decline during the maturation phase (1, 2). Generally, macrophages are divided into M1 (classical activation) and M2 (alternative activation) phenotypes. M1 macrophages phagocytize pathogens and necrotic tissues and secrete inflammatory factors, while M2 macrophages help to relieve inflammation and promote angiogenesis by releasing anti-inflammatory factors and growth factors. Diabetic patients experience wounds characterized by a prolonged inflammatory state, with overactivated M1-like macrophages and elevated levels of proinflammatory factors with inadequately activated M2 phenotypes and impaired expression of growth factors (3, 4). Because macrophages are an important target for the treatment of chronic diabetic wounds, studying the regulation of macrophage polarization may uncover therapeutic avenues to accelerate wound repair in diabetes.

miRNAs, 20–24 nucleotide (nt) non-coding RNAs, have the ability to regulate gene expression and the network of cellular processes. One miRNA can regulate the expression of multiple target genes, while genes involved in the same physiological or pathological process may be regulated by a single or a few miRNAs. Accordingly, miRNAs have advantages over conventional genes when used as therapeutic targets in diseases (5). Many studies have determined that miRNAs are important in regulating macrophage polarization (6, 7). Furthermore, M1 and M2 phenotypes play distinctive roles in the progression of refractory diabetic wounds. Thus, miRNAs might affect the repair of diabetic wounds by regulating the conversion between M1 and M2 macrophages.

In the present study, we determined the miRNA expression profiles in different stages of wound healing using Illumina sequencing and real-time quantitative reverse transcript polymerase chain reaction (RT-qPCR) techniques. Furthermore, overexpression of mmu-miR-145a-5p using mimics inhibited M1 polarization and promoted M2 polarization *in vitro*. Administration of miR-145a-5p mimics during the initiation of the repair phase also significantly accelerated wound healing in diabetic mice.

## Materials and Methods

### Animals

BKS-Lepr^em2Cd479^/Nju (db/db) diabetic mice (male, 8–10-week-old) were purchased from the Peking University Experimental Animal Center (Beijing, China). The measured blood glucose value of the db/db mice was 23.86 ± 2.07 mmol/L while the normal was ~6 mmol/L, indicating that the diabetic mouse models were successfully established. Normal C57BL/6J mice (male, 8–10-week-old) were purchased from Weitonglihua Co., Ltd. (Beijing, China). All animals were maintained in an animal facility, where the temperature was 22 ± 2°C and the humidity 55 ± 10% on a 12 h light-dark cycle. Food and water were freely available.

### Wound Healing Model

Mice were anesthetized with pentobarbital (i.p., 80 mg/kg). The dorsal surface was then shaved and cleaned with 75% ethanol. Two round wounds with a diameter of 5 mm were made on each side of the midline of the shaved dorsum using a punch biopsy tool (Miltex, USA). The wounds were left undressed, and all mice were housed separately thereafter. The wounds were imaged using a high-resolution camera, the sizes of which were calculated using ImageJ software (National Institutes of Health, USA). Biopsies were harvested on postoperative days 0, 1, 3, 5, 7, 15, 19, and 21.

### RNA Isolation and Library Preparation

During the inflammation, tissue formation, and tissue reconstruction phases of skin wound healing in control mice, namely, at 0, 1, 3, and 7 days post-operation, the wounds and a 5 mm unwounded skin border were harvested and stored at −80°C until further processing. Total RNA was isolated using the miRNeasy Serum/Plasma Kit (Qiagen, USA). The A (260/280) absorbance ratio of isolated RNA was 1.8–2.0, while the A (260/230) absorbance ratio was >1.6. Integrity of total RNA was determined using formaldehyde denaturing gel electrophoresis. The libraries were constructed from total RNA using the Illumina TruSeq Small RNA Sample Preparation kit (Illumina, USA). Single-stranded cDNA was created by reverse transcription reactions with the ligation products. The cDNA was amplified by PCR. Finally, Illumina sequencing technology was used to sequence these prepared samples.

### Illumina Sequencing and RT-QPCR Validation

Raw sequences were processed using the Illumina pipeline program. Clean reads were filtered, and contaminated reads were removed. Secondary structure prediction of individual miRNAs was performed using Mfold software. Clean sequence reads were mapped using miRBase 20.0, and a mismatch of 1–2 nucleotide bases was allowed. All data were transformed to log base 2. Differences between the samples were analyzed using chi-square and Fisher's exact tests. miRNAs with a fold difference N2.0 and *P* < 0.05 were considered statistically significant. miRNAs with high expression abundance and significant differences (fold change >4) in each phase of skin trauma were verified using RT-qPCR with Applied Biosystems TaqMan^TM^ MGB (minor groove binder) probes (Thermo Fisher Scientific, Waltham, MA, USA). The experiment was performed in strict accordance with the reagent instructions. All samples were analyzed in triplicate. The specificity of each PCR product was validated by melting curve analysis at the end of PCR cycles, and the cycle threshold (Ct) value was defined as the number of cycles required for the fluorescent signal to reach the threshold. Relative expression levels of miRNAs in wound samples were calculated using the 2–ΔΔCt formula, where ΔΔCt = [Ct (target, test) – Ct (ref, test)] – [Ct (target, calibrator) – Ct (ref, calibrator)]. All primers used were obtained from Invitrogen (Carlsbad, CA, USA).

### Transfection of miR-145a-5p Mimics and Negative Control

RAW 264.7 macrophage cells were grown in Roswell Park Memorial Institute medium (RPMI) 1640 (Gibco, USA) containing 10% fetal bovine serum (FBS, Gibco), penicillin (100 U/mL), and streptomycin (100 μg/mL) at 37°C in a 5% CO_2_ atmosphere. Macrophage polarization was induced with 1 μg/ml lipopolysaccharide (LPS, Sigma, L2880) or 20 ng/ml interleukin-4 (IL-4, Proteintech, 214-14). The cells were then transfected with 4 nM miR-145a-5p mimics (4464066, MC11480, Thermo Fisher Scientific) and negative control (4464059, Thermo Fisher Scientific) using Lipofectamine RNAiMAX (Invitrogen) according to the instructions and were harvested 48 h later. Three repeats were performed for each group.

For the *in vivo* study, miR-145a-5p mimics and negative control were diluted to 4 nM in autoclaved phosphate-buffered saline (PBS) and injected subcutaneously around the wound at a volume of 4 μL in each quadrant. The injections were repeated every 3 days for 2 weeks.

### Quantitative Real-Time PCR

At predetermined time points, total RNA was isolated from skin tissues and RAW 264.7 macrophage cells using TRIzol reagent (Invitrogen). Thereafter, complementary DNAs (cDNAs) were synthesized using the RevertAid First Strand cDNA Synthesis Kit (Thermo Fisher Scientific) according to the manufacturer's instructions. The resulting cDNAs were amplified with 40 cycles by RT-qPCR using Power SYBR^@^ Green PCR Master Mix (Thermo Fisher Scientific). Each sample was analyzed three times and normalized to β-actin as the internal control. Primer sequences for RT-qPCR are listed in Table 1.

**Table 1:** Primers for RT-qPCR analysis in mice.

**Genes**	**Forward primer (5^′^-3^′^)**	**Reverse primer (5^′^-3^′^)**
TNF-α	CAGGCGGTGCCTATGTCTC	CGATCACCCCGAAGTTCAGTAG
IL-1α	CGAAGACTACAGTTCTGCCATT	GACGTTTCAGAGGTTCTCAGAG
TGF-β2	TCGACATGGATCAGTTTATGCG	CCCTGGTACTGTTGTAGATGGA
Smad5	GAGCCATCACGAGCTAAAACC	ACTGGAGGTAAGACTGGACTCT
CD86	GGTGGCCTTTTTGACACTCTC	TGAGGTAGAGGTAGGAGGATCTT
CD206	CTCTGTTCAGCTATTGGACGC	CGGAATTTCTGGGATTCAGCTTC
Arg-1	TGACTGAAGTAGACAAGCTGGGGAT	CGACATCAAAGCTCAGGTGAATCGG
β-Actin	GGCTGTATTCCCCTCCATCG	CCAGTTGGTAACAATGCCATGT

### Histological Analysis and Immunohistochemistry

The wounds and a 5 mm unwounded skin border were harvested from mice and processed for histological analysis. Wound widths were measured as the distance between wound margins. Wound areas were determined using image analysis (measured below the clot and above the panniculus muscle). Re-epithelialization was defined as the percentage of distance migrated by the neoepidermis compared to the upper wound width. Immunohistochemistry analysis was performed on formalin-fixed, paraffin-embedded 5-μm sections. The primary antibodies used in this study were against keratin 14 (K14, 1:1000, Covance, Princeton, NJ, USA), Ki67 (1:1000, Covance) and CD206 (1:500, Novus Biologicals, CO, USA). To quantify CD206 expression, ImageJ 1.50d (USA) was used to analyze the integrated optical density.

### Bioinformatics Analysis of Downstream Target Genes of mmu-miR-145a-5p

To predict target genes of mmu-miR-145a-5p, TargetScan (5.0), miRanda (3.3a), and PITA (1.0) analyses were conducted and the default thresholds were targetscan_score ≥50, miranda_Energy < −10, and ddG < −5. Thereafter, the intersections of the three databases were selected as candidate downstream miRNAs of mmu-miR-145a-5p for further analysis. Functional enrichment analysis of the target genes of miR-145a-5p was conducted using clusterProfiler with Gene Ontology.db. (GO) and the Kyoto Encyclopedia of Genes and Genomes.db. (KEGG). Significance was assessed using hypergeometric tests and Benjamini-Hochberg correction, with a significance threshold of *p* < 0.05 (8, 9).

### Statistical Analysis

All results are presented as the mean ± SD. All experiments were performed with a minimum of three independent replicates. One-way ANOVA followed by Bonferroni *post-hoc* tests were performed to analyze multiple groups and unpaired Student's *t*-test was performed to analyze two parallel groups, using SPSS software (IBM, Armonk, NY, USA). Differences were considered significant at two-sided *p* < 0.05.

## Results

### Differentially Expressed miRNAs During Wound Healing in Mice

**Figure 1:**
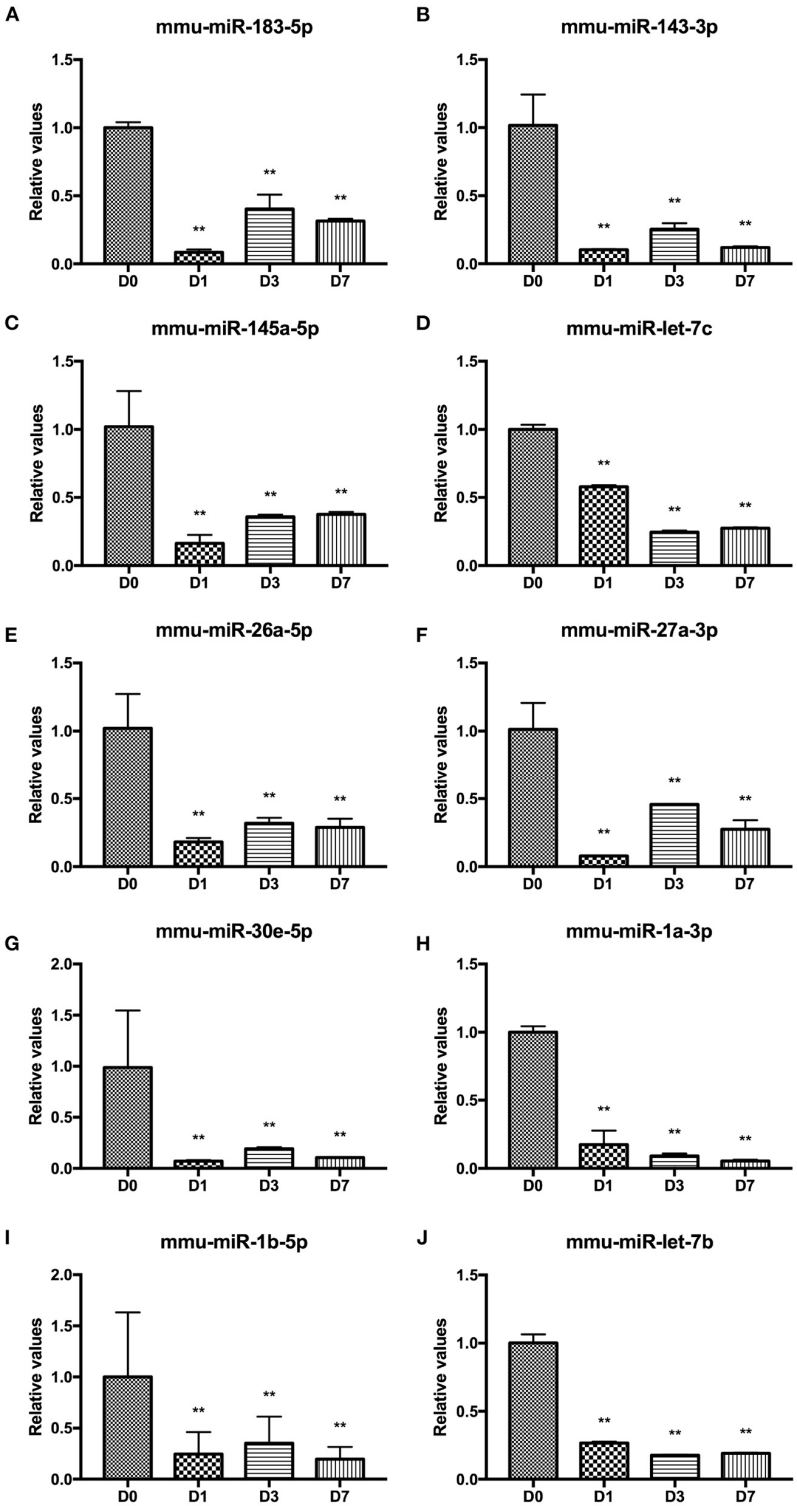
Differentially expressed miRNAs at different stages of wound healing. **(A)** mmu-miR-183a-5p. **(B)** mmu-miR-143-3p, **(C)** mmu-miR-145a-5p. **(D)** mmu-miR-let-7c. **(E)** mmu-miR-26a-5p. **(F)** mmu-miR-27a-3p. **(G)** mmu-miR-30e-5p. **(H)** mmu-miR-1a-3p. **(I)** mmu-miR-1b-5p. **(J)** mmu-miR-let-7b. **P < 0.01 vs. control (D0).

High-throughput miRNA sequencing (miRNA-Seq, 10 million reads/sample) was performed on unharmed skin tissues (D0) and during inflammation (postinjury day 1, D1), tissue formation (postinjury day 3, D3) and tissue reconstruction (postinjury day 7, D7) phases during wound healing in normal male C57BL/6J mice. Table 2 shows the total number of differentially expressed miRNAs between different groups (fold change ≥2 and *P* ≤ 0.01). The miRNAs with high expression abundance and significant differences (a fold change >4) in each phase of skin trauma were verified using real-time PCR with the probe method. The verification results for 10 miRNAs were consistent with the sequencing results, and expression levels of mmu-miR-183a-5p, mmu-miR-143-3p, mmu-miR-145a-5p, mmu-miR-let-7c, mmu-miR-26a-5p, mmu-miR-27a-3p, mmu-miR-30e-5p, mmu-miR-1a-3p, mmu-miR-1b-5p, and mmu-miR-let-7b were significantly downregulated 1–7 days after trauma (Figure 1). These results suggest that miRNAs participate in the regulation of different stages of wound healing and might be potential therapeutic targets in diseases related to abnormal wound repair.

**Table 2:** Total number of differentially expressed miRNAs (fold change ≥2 and *P* ≤ 0.01).

**DEG sample**	**Total miRNAs**	**Down regulated miR NO**.	**Up regulated miR NO**.	**Total DEmiR**
D1 vs. D0	1141	48	60	108 (9.47%)
D3 vs. D0		118	97	215 (18.84%)
D7 vs. D0		110	98	208 (18.23%)
D3 vs. D1		107	66	173 (15.16%)
D7 vs. D1		100	97	197 (17.27%)
D7 vs. D3		25	46	71 (6.22%)

### An Excessive Inflammatory Response and Insufficient Growth Factor Secretion During the Formation Phase of Skin Trauma Causes Delayed Wound Healing in Diabetic Mice

**Figure 2:**
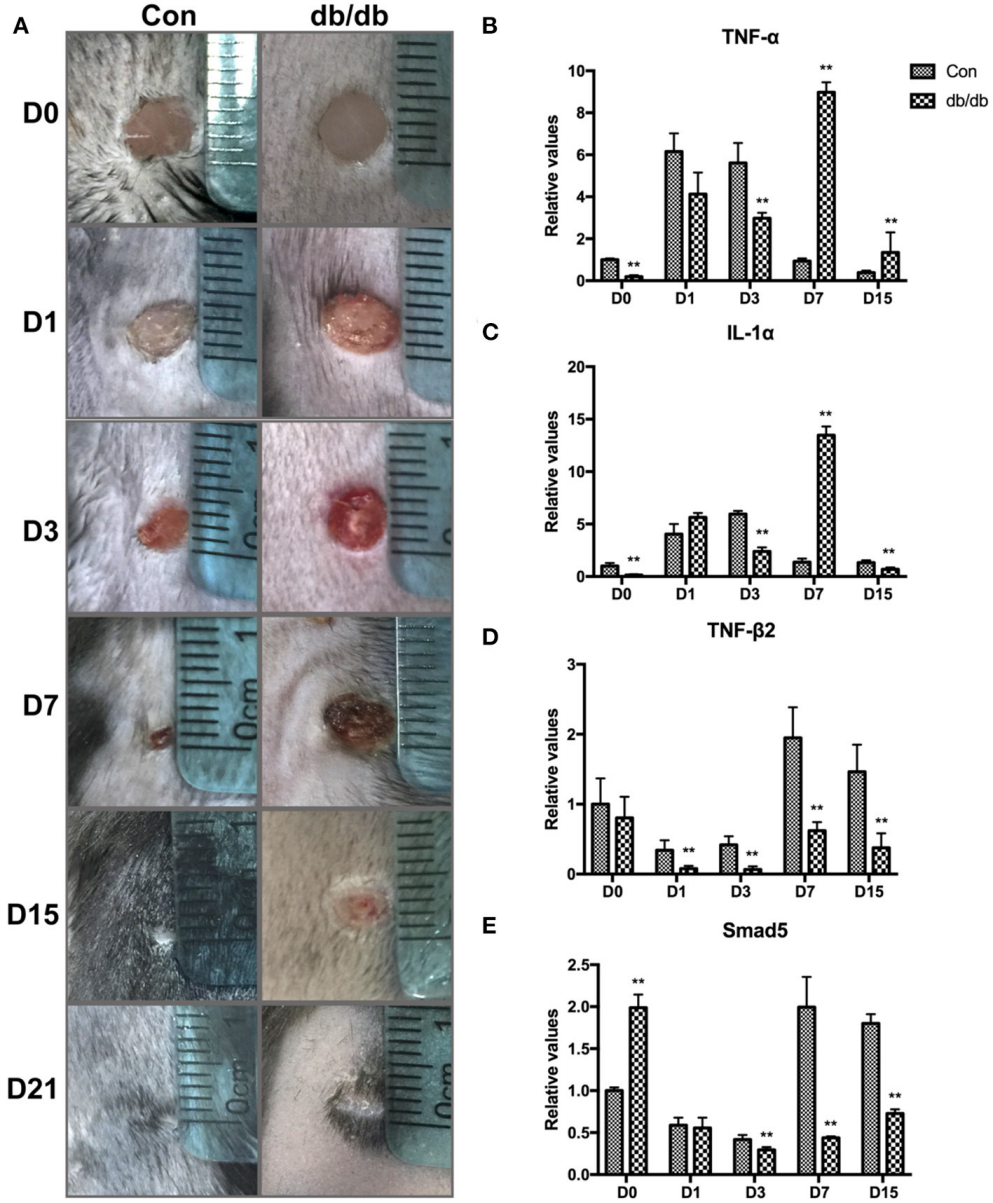
Changes in wound size and the expression of proinflammatory and growth factors in diabetic wound healing. **(A)** Representative images of excisional wounds in db/db and control mice on days 0, 1, 3, 7, 15, and 21. **(B–E)** Expression of proinflammatory and growth cytokines in the db/db and control mice on days 0, 1, 3, 7, and 15. **P < 0.01 vs. the corresponding control (Con) group at the same time points.

Next, we examined the changes in wound repair and the inflammatory state in diabetic mice. The wound closure speed in the db/db mice was dramatically lower than that in the control group (Figure 2A). We also determined cytokine expression in wound tissues and found that expression levels of the proinflammatory cytokines tumor necrosis factor alpha (TNF-α) and interleukin-1alpha (IL-1α) in db/db mice were downregulated on days 1 and 3 and elevated on day 7 post-injury, suggesting insufficient activation of M1 macrophages during the inflammatory stage of the wounds but overreaction during the tissue formation stage (Figures 2B,C). Quite a number of studies have reported the abnormal inflammation in chronic diabetic wound, which is characterized by the upregulation of pro-inflammatory cytokines, such as TNF-a, IL-1α, IL-1β, and IL-6 (10–13). Shang et al. found the increased M1-like macrophages on d7 in diabetic mice, which were believed to be closely related to the expression of pro-inflammatory factors (14). In addition, the growth factors transforming growth factor beta 2 (TGF-β2) and Smad5 in the db/db mice decreased significantly at different time points post-injury, indicating reduced polarization of M2 macrophages in all stages of diabetic wound healing (Figures 2D,E). Collectively, the failure of transformation from M1 macrophages to M2 phenotypes during the tissue formation stage might be an important mechanism of delayed wound healing in diabetes mellitus.

### miR-145a-5p Promotes Macrophage Polarization Toward M2 *in vitro*

**Figure 3:**
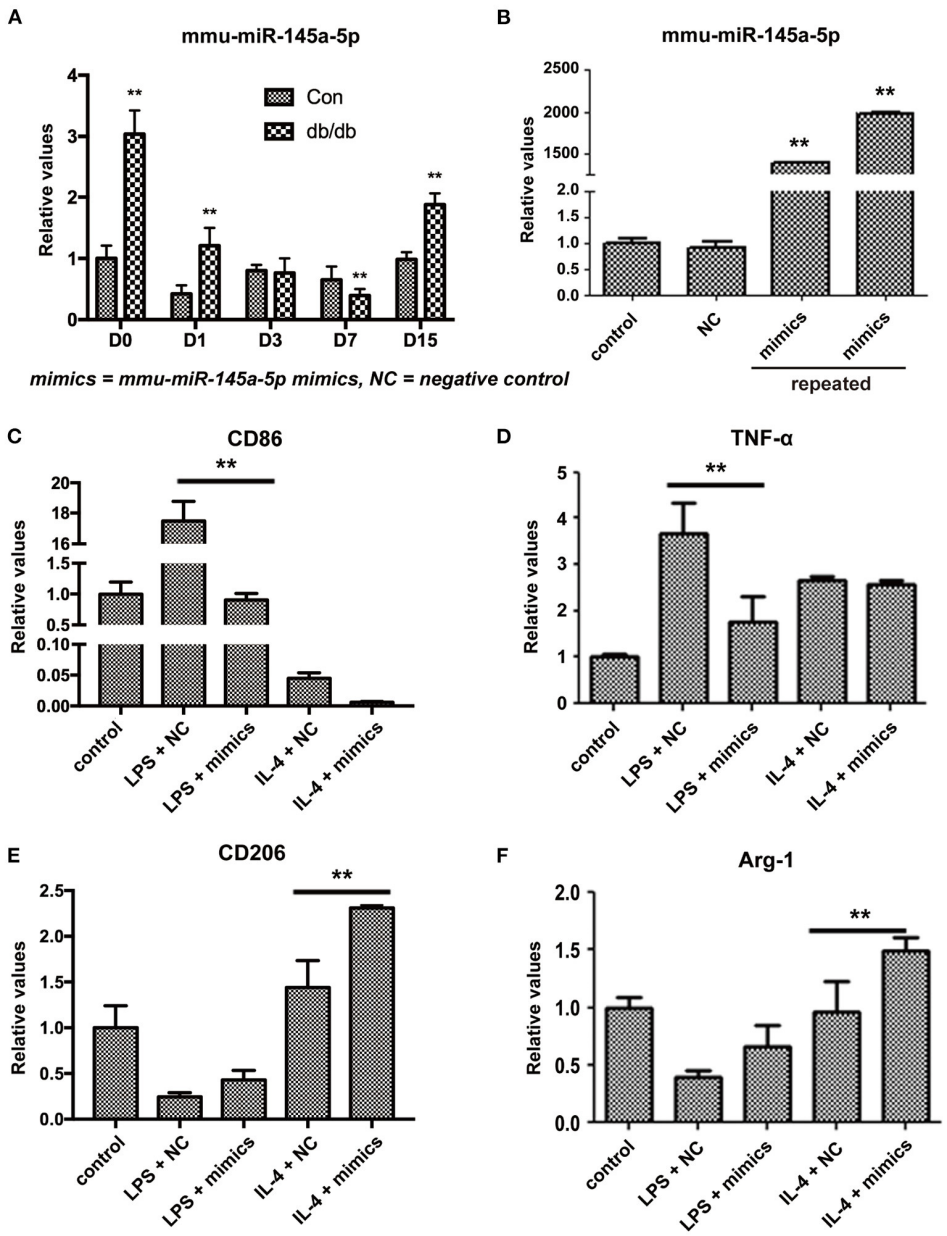
Influence of miR-145a-5p on macrophage polarization in RAW 264.7 cells. **(A)** Expression of miR-145a-5p in db/db and control mice on postoperative days 0, 1, 3, 7, and 15. **(B)** Expression of miR-145a-5p in RAW 264.7 macrophages 24 h after the addition of miR-145a-5p mimics or negative control miRNA. **(C–F)** Expression of CD86, TNF-α, CD206 and Arg-1 in RAW 264.7 macrophages pretreated with LPS or IL-4 in combination with miR-145a-5p mimics/negative control miRNA. **P < 0.01 vs. the corresponding control group.

miR-145a-5p was one of the differentially expressed miRNAs during wound healing identified by microarray and RT-qPCR. Many studies have reported the regulatory effects of miR-145a-5p on cell differentiation, such as myoblasts, adipocytes, and microglia (15–17). Whether miR-145a-5p affects the polarization of macrophages in diabetic wound healing greatly excited our interest. First, we studied the expression of miR-145a-5p in wound tissues of db/db mice and found that expression levels of miR-145a-5p increased on days 0, 1, and 15 but were significantly decreased on day 7 after the operation (Figure 3A). miR-145a-5p was involved in the regulation of inflammation (18), and the higher expression level of miR-145a-5p in db/db mice than in control mice on day 0 might be explained by diabetic microangiopathy and inflammation. These results suggest that miR-145a-5p is involved in the pathophysiological process of diabetic wound healing.

To further clarify the effects of miR-145a-5p on macrophage polarization, we transfected miR-145a mimics or the related negative control (NC) in RAW 264.7 macrophage cells pretreated with LPS or IL-4. Transfection of miR-145a-5p mimics into RAW 264.7 cells increased the expression levels of miR-145a-5p > 1000-fold compared to that of the NC group (Figure 3B). Generally, CD86 and tumor necrotic factor-α (TNF-α) are used as biomarkers generated by M1 macrophages, while CD206 and arginase 1 (Arg-1) are accepted markers of the M2 phenotype (19, 20). miR-145a-5p mimic transfection dramatically downregulated expression of CD86 and TNF-α in RAW 264.7 macrophages pretreated with LPS, suggesting that miR-145a-5p abolished M1 polarization induced by LPS (Figures 3C,D). On the other hand, miR-145a-5p overexpression caused increased expression of CD206 and Arg-1 in macrophages pretreated with LPS or IL-4, indicating the roles played by miR-145a-5p in facilitating M2 polarization (Figures 3E,F). These results demonstrate that miR-145a-5p promotes macrophage polarization toward M2.

### miR-145a-5p Mimics Accelerate Wound Healing in db/db Mice

**Figure 4:**
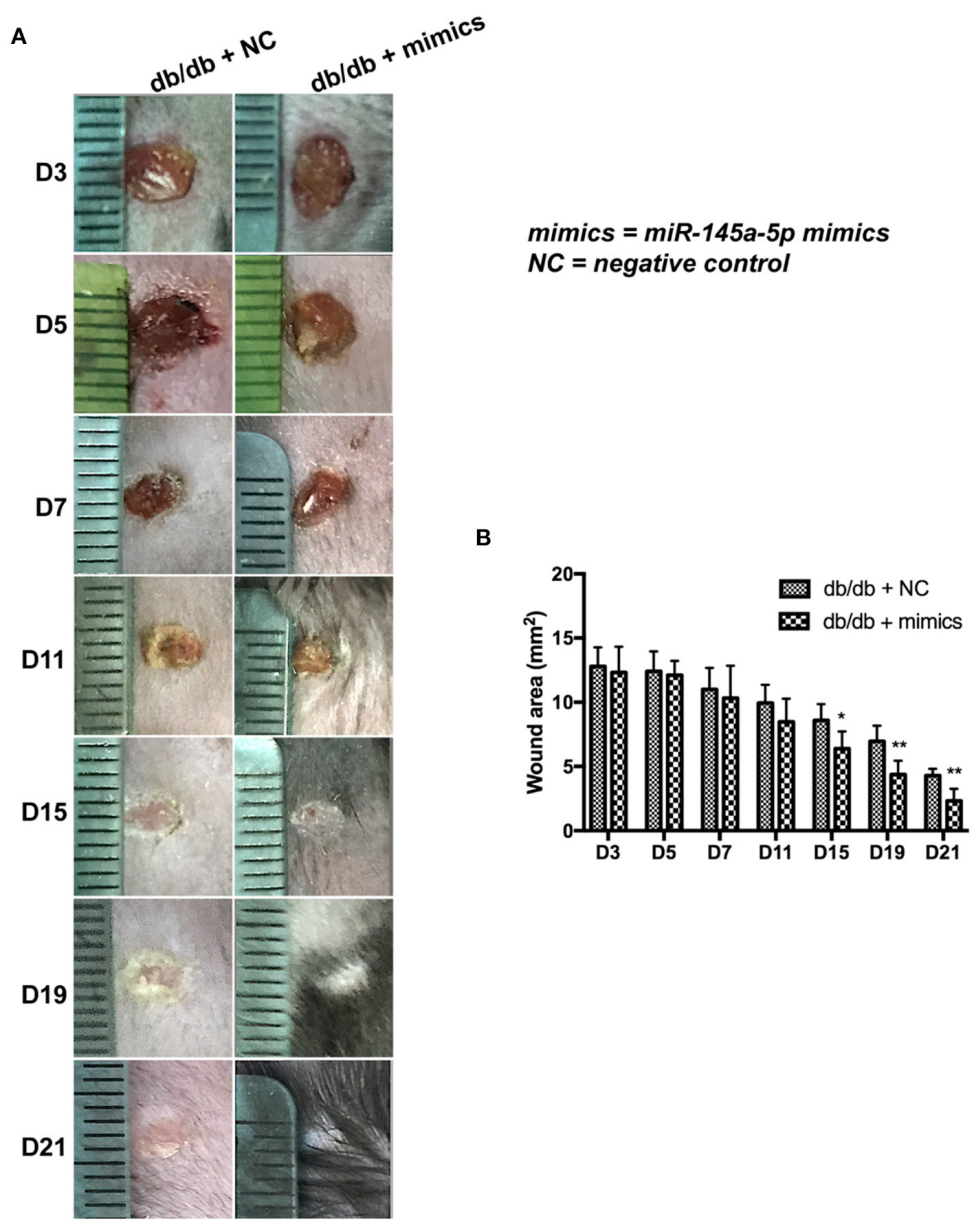
Effect of miR-145a-5p on wound healing size in diabetic mice. **(A)** Representative images of excisional wounds of db/db mice treated with miR-145a-5p mimics or the corresponding negative control. **(B)** Wound size measurement. *P < 0.05, **P < 0.01 vs. the corresponding control group.

**Figure 5:**
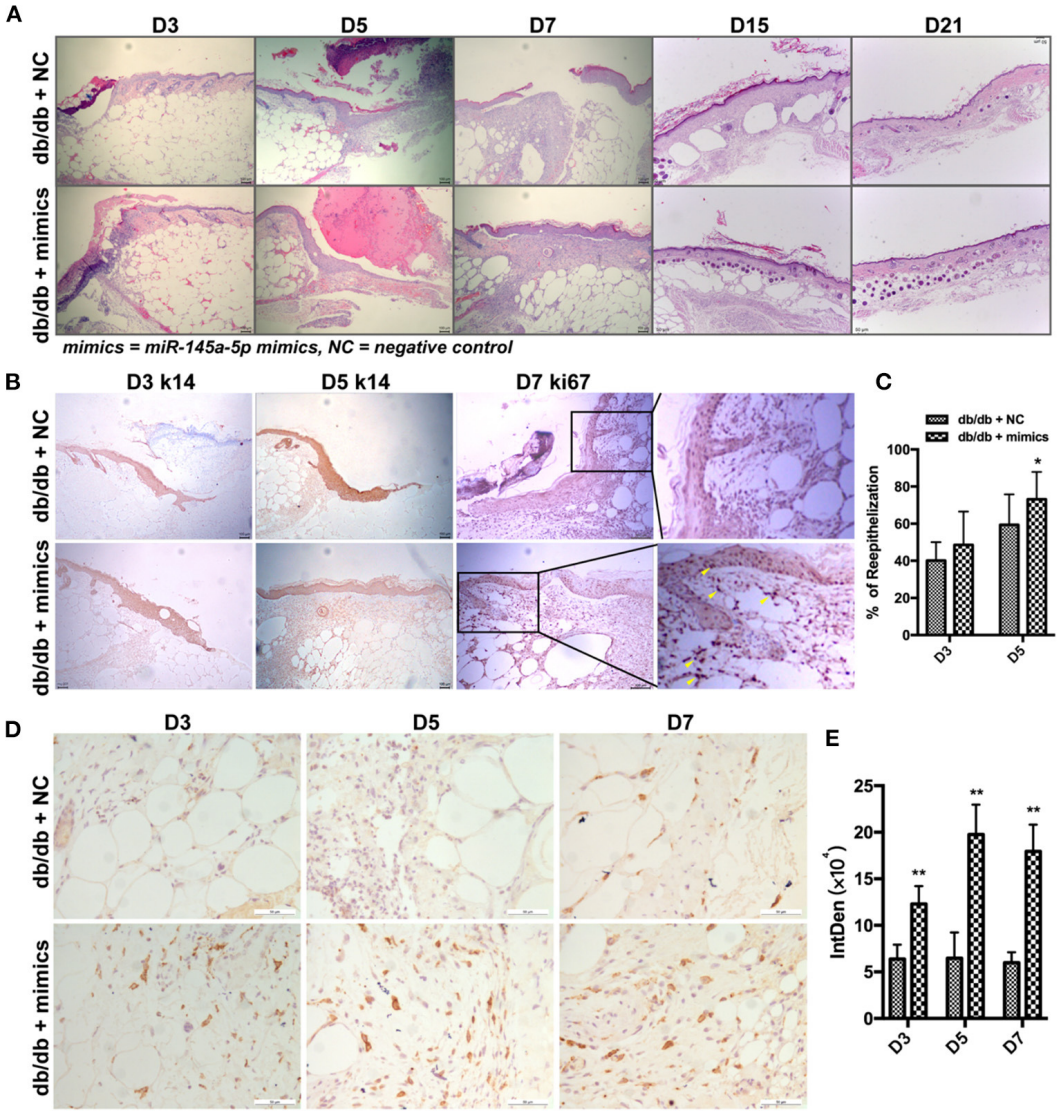
Effect of miR-145a-5p on keratinocyte reepithelialization of wound healing in diabetic mice. **(A)** Representative H&E stained images of excisional wounds in db/db mice treated with miR-145a-5p mimics or negative control on days 3, 5, 7, 15, and 21. **(B)** Representative immunohistochemistry (IHC) stained images of K14 and Ki67 in wounds of db/db mice treated with miR-145a-5p mimics or negative control on days 3, 5, and 7. Ki67^+^ cells are indicated with yellow arrows. **(C)** Statistical analysis of epithelialization ratio. **(D)** Representative IHC images of CD206 in diabetic wounds treated with miR-145a-5p mimics or negative control on days 3, 5, and 7. **(E)** Statistical analysis of the integrated optical density of CD206 from D. *P < 0.05 and **P < 0.01 vs. the corresponding control group at the same time points.

miR-145a-5p mimics were administered locally to assess their therapeutic effect on diabetic trauma. The wound closure rate in db/db mice treated with miR-145a-5p mimics was dramatically higher than that in the control group (Figures 4A,B). Histological analysis revealed that wounds in the miR-145a-5p mimic group healed at a faster rate than those in the control group (Figure 5A). Immunohistochemistry of K14 showed that the neoepithelial lengths were longer in the healing skin of db/db mice transfected with miR-145a-5p mimics than in those treated with negative control miRNAs, and enhanced expression of Ki67 on postoperative day 7 suggested an increased number of proliferative cells during the tissue formation stage when miR-145a-5p was overexpressed (Figures 5B,C). Importantly, miR-145a-5p mimics increased the expression of CD206 in diabetic wounds, suggesting the ability of miR-145a-5p to promote M2 polarization *in vivo* (Figures 5D,E). These results show that overexpression of miR-145a-5p accelerates diabetic wound healing and increases M2 phenotypes in diabetic wounds.

### Go and KEGG Functional Enrichment Analysis of miR-145a-5p Downstream Genes

**Figure 6:**
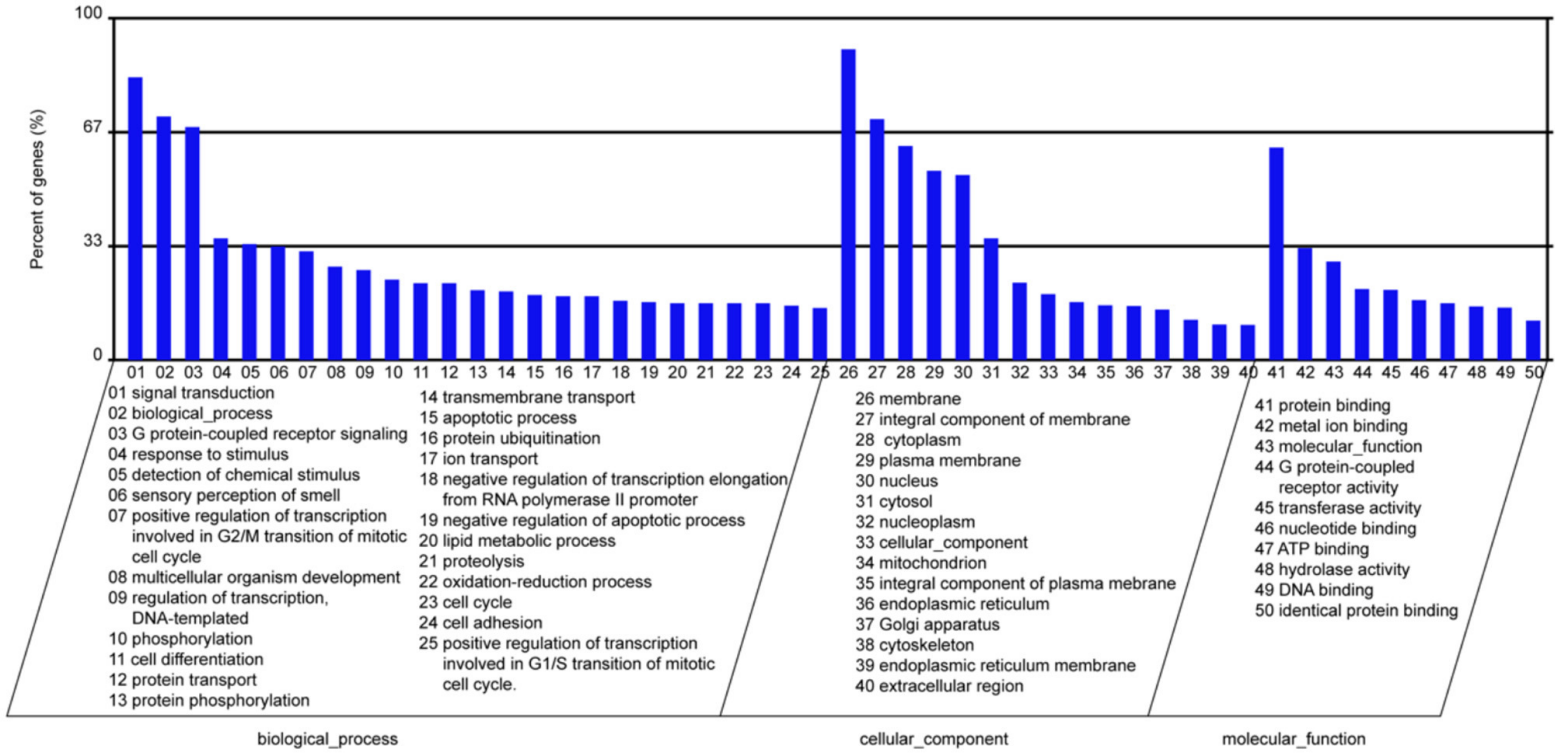
Functional enrichment analysis of the predicted downstream genes of miR-145a-5p.

In the present study, we screened the downstream genes of miR-145a-5p using 3 databases, including TargetScan, miRanda, and PITA. Subsequently, 2,473 target genes of miR-145a-5p predicted by all three databases were selected. GO and KEGG functional enrichment analyses revealed that miR-145a-5p is involved in regulating many biological processes, including signal transduction, cell differentiation, apoptotic processes, etc. (Figure 6).

## Discussion

The normal wound healing response is characterized by progression from clot formation to an inflammatory phase, a repair phase, and finally remodeling. Macrophages have been verified to be important in the inflammatory phase of tissue repair, where their dynamic plasticity allows these cells to mediate both tissue-destructive and tissue-reparative functions (10). In diabetic chronic wounds, an extended inflammatory phase stemming from macrophage functional disturbance halts this progression (21, 22). The phagocytosis rate of M1 macrophages in the diabetic state is >5 times slower than normal, which may lead to slower clearance of wound necrotic tissue and apoptotic cells and sustained release of inflammatory factors. The blocked transformation from the M1 to M2 phenotype and the insufficient secretion of anti-inflammatory factors and growth factors lead to failure of normal tissue formation and eventually prolonged healing in diabetic mellitus (23, 24). Some studies have attempted to treat diabetic trauma by regulating the macrophage-mediated inflammatory response and have achieved positive effects, suggesting macrophages as a potential therapeutic target in refractory wounds in diabetic mellitus (25–30). Our research group has been committed to investigating wound healing mechanisms and therapy and previously found that knockdown of casein kinase 2-interacting protein 1 (CKIP-1) in macrophages inhibited M1-type activation and slowed the wound healing rate (31). However, innovative methods still need to be explored to regulate the macrophage response during wound healing. In this study, we identified miR-145a-5p as one of the differentially expressed miRNAs between db/db diabetic mice and controls. miR-145a-5p has been demonstrated to play important regulatory roles in cell differentiation and polarization, such as myoblasts, adipocytes, and microglia (15–17). Therefore, the regulation of miR-145a-5p on the macrophage response and its effect on diabetic wound healing aroused our interest.

miRNAs are a class of endogenous RNAs of ~22 nucleotides in length produced by dicer 1, ribonuclease III (Dicer) processing single-stranded RNA precursors of ~70–90 bases (32). miRNAs bind to the corresponding 3′ untranslated region (3′UTR) of the target mRNA through complete or incomplete pairing, preventing its translation or disrupting its stability to regulate gene expression (33). miRNAs regulate 30% of all human genes and have extensive regulatory functions in life activities and profound effects on gene expression, growth, and behavior (34, 35). miRNAs are widely involved in various skin wound healing processes (36). miR-125b regulates leukocyte aggregation and activation by inhibiting TNF-α expression (37). miR-125b inhibits keratinocyte proliferation by downregulating fibroblast growth factor receptor 2 (FGFR2) (38). In ischemic trauma, hypoxia in the wound site and high miR-210 expression reduce keratinocyte proliferation by inhibiting E2F transcription factor 3 (E2F3) expression (39). miR-483-3p depresses keratinocyte proliferation and migration by downregulating mitogen-activated protein kinase-activated protein kinase 2 (MAPKAPK2, or MK2), Ki67, and Yes-associated protein 1 (YAP1) (40). miR-203 regulates keratinocyte proliferation and migration at the wound margin by affecting RAN and Ras association (RalGDS/AF-6) and pleckstrin homology domain 1 (RAPH1) (41). miR-205 promotes keratinocyte migration by inhibiting inositol polyphosphate phosphatase-like 1 (SHIP2) (42, 43). miR-21 downregulates tissue metalloproteinase inhibitor 3 (TIMP3) and T-cell lymphoma invasion and metastasis 1 (TIAM1) to promote keratinocyte migration (44). miR-21 is elevated in the skin of diabetic mice and regulates wound contraction and collagen deposition (45, 46). miR-27b inhibits mouse mesenchymal stem cell migration to burn sites by silencing C-X-C motif chemokine ligand 12 (SDF-1A), which slows wound healing (47). Members of the miR-199 family are involved in the regulation of wound healing through the AKT/mammalian target of rapamycin (mTOR) pathway (48). let-7c overexpression inhibits the M1 macrophage phenotype and promotes the M2 phenotype (49). LPS-pretreated mesenchymal cells secrete let-7b from exosomes, promoting macrophage secretion of anti-inflammatory factors and targeting M2 differentiation, while miR-130b inhibits M2 macrophage activation by targeting peroxisome proliferator-activated receptor gamma (PPARγ) (50). In conclusion, miRNAs are intimately involved in the regulation of wound healing. In this study, we screened out miRNAs related to the traumatic inflammatory response, tissue formation, and tissue remodeling by chip and finally verified 10 differentially expressed miRNAs (high expression and significant difference) using RT-qPCR. Among these miRNAs, miR-145a-5p was selected for further study due to its potential to regulate macrophage polarization.

In this study, we found that local application of miR-145a-5p dramatically promoted wound healing in db/db mice. K14 is an intermediate filament protein that is mainly expressed in epithelial cells, which are generally used as a research biomarker of epidermal skin (51). Ki67 is universally expressed among proliferating cells, but is absent from quiescent cells (52). miR-145a-5p was found to facilitate diabetic wound healing, of which the indicators were chiefly the accelerated repair, the larger K14 positive area, as well as the increased Ki67 positive cells. Macrophage dysfunction is an important characteristic of delayed wound healing in diabetes mellitus (21, 22). In this study, functional experiments revealed that miR-145-5p overexpression inhibited M1 macrophage polarization while promoting M2 polarization in RAW 264.7 macrophage cells. Importantly, the overexpression of miR-145-5p increased the number of M2 macrophages (CD206^+^) in diabetic wound. It has been demonstrated that macrophages of M2 facilitate the resolution of inflammation and promote tissue remodeling by releasing growth cytokines and anti-inflammatory cytokines (10, 11). Therefore, the polarization of macrophage toward the M2 phenotype mediated by miR-145a-5p might be an important mechanism responsible for its therapeutic effects on diabetic wound repair. Additionally, the bioinformatics analysis of the downstream miRNAs of miR-145a-5p suggested that miR-145a-5p was involved in the regulation of signal transduction, cell differentiation, apoptosis, cell cycle, lipid metabolic process, etc. Wang et al. found that miR-145a-5p regulated microglial polarization and the production of inflammatory cytokines, in which the NF-κB pathway might be involved (17). Chen et al. reported that miR-145a-5p modulated the polarization of M2 macrophage by targeting PAK7 and regulating β-catenin signaling in hyperlipidemia (53). Moreover, miR-145a-5p regulates the differentiation of myoblasts, adipocytes, and T cells (15, 16, 54). However, the underlying mechanisms by which miR-145a-5p regulates macrophage polarization have not been elucidated. More work is needed to verify the downstream genes and related signaling pathways of miR-145a-5p in regulating macrophage polarization.

The other differentially expressed miRNAs identified in our study, such as miR-183-5p, miR-143-3p, miR-let-7c, miR-26a-5p, miR-27a-3p, miR-30e-5p, and miR-let-7b, were mainly involved in the regulation of cellular proliferation and inflammation. The functions of these miRNAs were found to be chiefly as follows: First, miR-183-5p transferred to macrophages by exosomes promoted the secretion of proinflammatory cytokines (55); second, miR-143-3p participated in the regulation of LPS induced inflammation (56); third, miR-let-7c inhibited proinflammatory cytokine production in osteoarthritis and rheumatoid arthritis synovial fibroblasts (57); fourth, miR-26a-5p was capable of protecting against retinal neuronal impairment in diabetic mice by down-regulating phosphatase and tensin homolog (PTEN) (58); fifth, miR-27a-3p attenuated LPS-induced HK-2 cell apoptosis by downregulating the protein levels of TLR4 and NF-kappaB (59, 60); sixth, miR-30e-5p alleviated inflammation and cardiac dysfunction after myocardial infarction through targeting PTEN (61, 62); and seventh, miR-let-7b regulated the expression of inflammation-associated genes in monocytes, macrophages, and neutrophil (63, 64). The literature on the biological roles of miR-1a-3p and miR-1b-5p has been scanty. While these miRNAs might play important roles in the process of wound healing through the regulation of inflammation and cell proliferation, the effects of these identified miRNAs on wound healing as well as the underlying mechanisms remain to be further explored, where potential therapeutic targets, for instance, diabetic trauma, are expected to be pinpointed for chronic wound healing.

## Data Availability Statement

The datasets presented in this study can be found in online repositories. The names of the repository/repositories and accession number(s) can be found at: https://datadryad.org/stash/dataset/, https://doi.org/10.5061/dryad.n5tb2rbwx.

## Ethics Statement

The animal study was reviewed and approved by the Institutional Animal Care and Use Committee of Beijing Institute of Radiation Medicine.

## Author Contributions

YLi and LY: conceptualization. YH, HZ, YLiu, YY, CY, HY, YX, ZJ, and CH: methodology. YH and JW: formal analysis and writing—original draft preparation. YLi: writing—review and editing and supervision. LY: project administration and funding acquisition. All authors have read and agreed to the final version of the manuscript.

## Funding

This research was funded by the National Natural Science Foundation of China, grant number 81272104.

## Conflict of Interest

The authors declare that the research was conducted in the absence of any commercial or financial relationships that could be construed as a potential conflict of interest.

## Publisher's Note

All claims expressed in this article are solely those of the authors and do not necessarily represent those of their affiliated organizations, or those of the publisher, the editors and the reviewers. Any product that may be evaluated in this article, or claim that may be made by its manufacturer, is not guaranteed or endorsed by the publisher.
